# Information provided to patients with adolescent idiopathic scoliosis (AIS) at the first point of diagnosis in the hospital clinic: a survey of UK NHS scoliosis consultants

**DOI:** 10.1186/1748-7161-7-S1-O36

**Published:** 2012-01-27

**Authors:** J Bettany-Saltikov, D Martin, S Wellburn, P Van Schaik

**Affiliations:** 1 Institute of Health and Social Care, Middlesbrough, UK

## Purpose

The purpose of this survey was to determine what information is currently provided by NHS scoliosis centres in the UK at the point of first diagnosis of patients with AIS [1].

## Background

Service users` health information needs are very frequently not addressed in hospital clinics. The role of the patient as an active partner in health care is now widely accepted and providing information to patients is considered fundamental [2].

## Material and methods

An electronic survey was emailed to senior consultants at 30 key scoliosis centres in the UK. The survey covered questions relating to the most common questions asked by service users when first diagnosed, whether any written information was provided and who had written this and whether patients were referred to any relevant web sites.

## Results

The response rate was 47%. The most common questions asked by service users related to aetiology (22.5%) prognosis (42.6%) general treatment (16.8%) surgery (12.4%) and parental guilt (5.6%). 78.6% of consultants said that patients were provided with written information provided by a member of staff and written by the Scoliosis Association UK in 61.5% of cases. 92% of consultants referred patients to relevant web sites. Surgeons stressed the importance for information to be evidence-based, address patients anxieties and counselling needs, provide clear natural history information and address ways of contacting other patients with AIS who have or have not undergone surgery.

## Conclusions

AIS patients at the point of first diagnosis at hospital are provided with relevant information or referred to relevant web-sites in a significant number of scoliosis UK centres. Further studies are in progress to evaluate patients’ perceptions on the quality and format of information currently provided by NHS scoliosis centres.
